# Dataset on acute hyperglycemia in extensively burned patients and mice

**DOI:** 10.1016/j.dib.2018.11.089

**Published:** 2018-11-22

**Authors:** Jun Li, Jie Xu, Xing Zhang

**Affiliations:** a Department of Aerospace Medicine, Fourth Military Medical University, Xi’an 710032, China; b Department of Burns and Cutaneous Surgery, Xijing Hospital, Fourth Military Medical University, Xi’an 710032, China

## Abstract

A single center, prospective cohort study recruiting 107 burn patients and 62 control patients was conducted to characterize the early phase of acute hyperglycemia in patients with burns. A total of 1643 blood samples were collected, and blood glucose and serum proinflammatory cytokines were detected and analyzed. A mouse severe burn model was used to study the underlying mechanisms of acute hyperglycemia postburn. An expression profile of IL-1 receptor, serum IL-1β and pancreatic islet function were detected in both control mice and burned mice. The data in this article is associated with the research article published in Biochim Biophys Acta “Acute pancreatic beta cell apoptosis by IL-1β is responsible for postburn hyperglycemia: evidence from humans and mice” [1].

**Specifications table**

**Table:** 

Subject area	*Biology*
More specific subject area	*Burn injury*
Type of data	*Tables and figures*
How data was acquired	*ELISA, RT-PCR and Microscope*
Data format	*Analyzed*
Experimental factors	*Degree of burn injury, IL-1β injection*
Experimental features	*Human blood samples were collected from burn patients. Serum variables were detected by ELISA. A mouse severe burn model was induced in laboratory.*
Data source location	*Blood samples were collected from patients admitted to the Department of Burns and Cutaneous Surgery, Xijing Hospital, Fourth Military Medical University, China.*
Data accessibility	*Data are presented in this article.*
Related research article	J. Li, J. Xu, X. Qin, H. Yang, J. Han, Y. Jia, H. Zhu, L. Zhu, J. Li, W. Xie, D. Hu, X. Zhang, F. Gao, Acute pancreatic beta cell apoptosis by IL-1β is responsible for postburn hyperglycemia: evidence from humans and mice, Biochim Biophys Acta, (2018). In press [1].

**Value of the data**

• The data furnishes the scientific body with information on random blood glucose and its regulation in control and burn patients.
• The data describes changes in serum levels of major proinflammatory cytokines and their association with blood glucose postburn.
• The data on mice offers extra evidence on that serum IL-1β specifically induces pancreatic beta cell dysfunction.
• The data will foster further examination of the potential of IL-1β neutralization in glycemic control in patients with burns.

## 1 Data

**Fig. 1:**
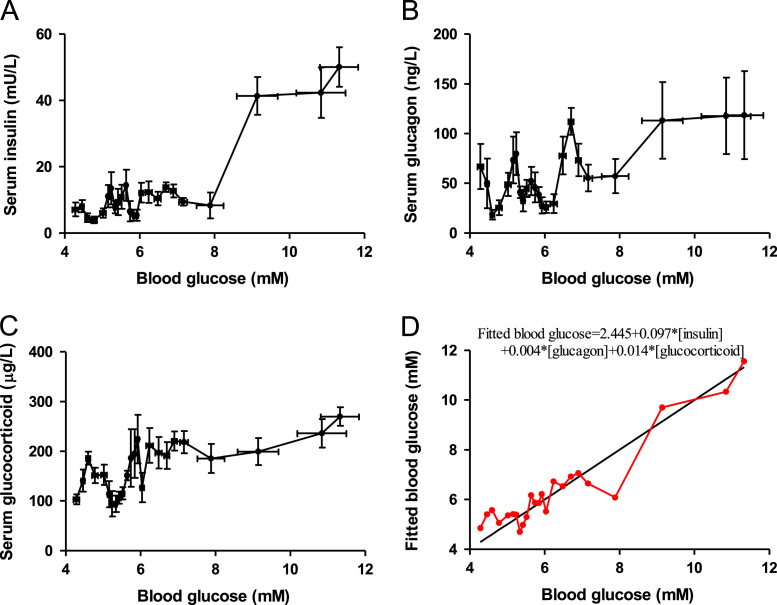
Linear regression for blood glucose with serum insulin, glucagon and glucocorticoid as covariates in control patients. A-C. Serum insulin (A), glucagon (B) and glucocorticoid (C) concentrations with respect to blood glucose. D. Linear regression for blood glucose with serum insulin, glucagon and glucocorticoid as covariates.

**Fig. 2:**
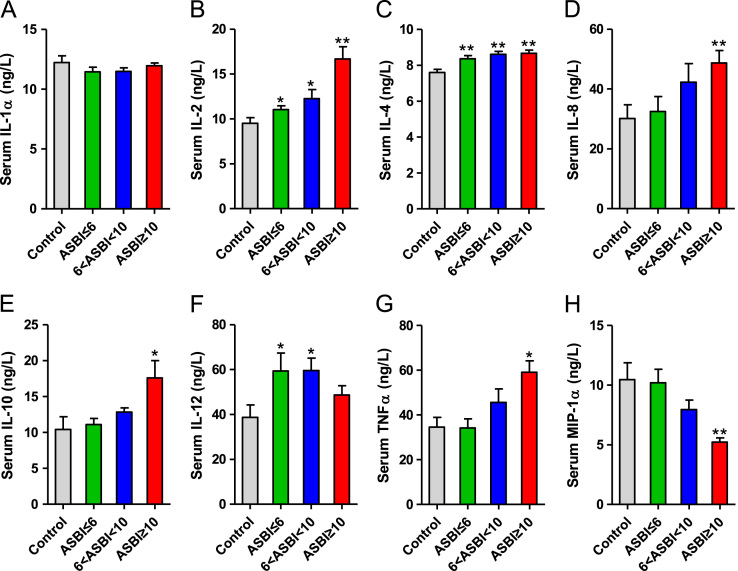
Levels of serum proinflammatory cytokines at 0–200 h postburn. A-H. Serum IL-1α (A), IL-2 (B), IL-4 (C), IL-8 (D), IL-10 (E), IL-12 (F), TNFα (G), and MIP-1α (H) concentrations in patients with burns. *, *P* < 0.05; **, *P* < 0.01.

**Fig. 3:**
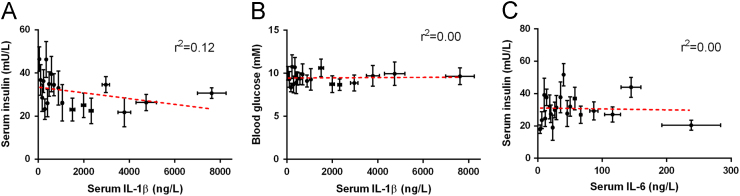
Correlations between serum insulin and serum IL-1β (*P* = 0.15) (A), blood glucose and serum IL-1β (*P* = 0.88) (B), and serum insulin and serum IL-6 (*P* = 0.87) (C).

**Fig. 4:**
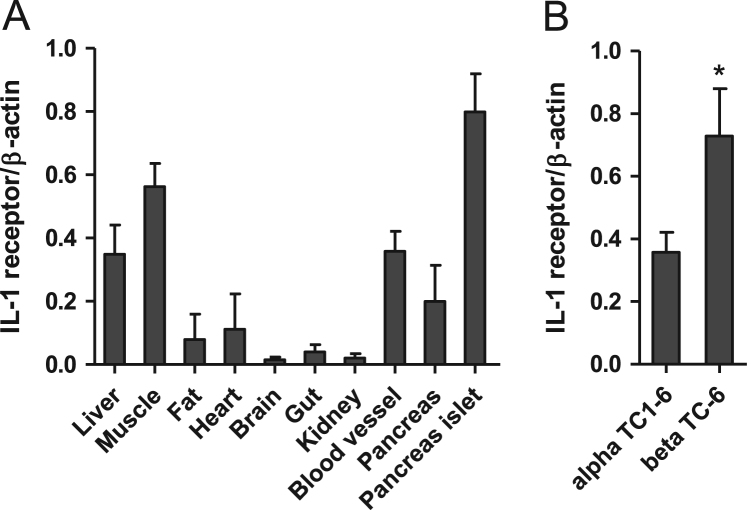
A tissue expression profile of IL-1 receptor. A. A tissue expression profile of IL-1 receptor in mice. B. IL-1 receptor expressions in beta TC-6 cells and alpha TC1–6 cells. *n* = 4, *, *P* < 0.05.

**Fig. 5:**
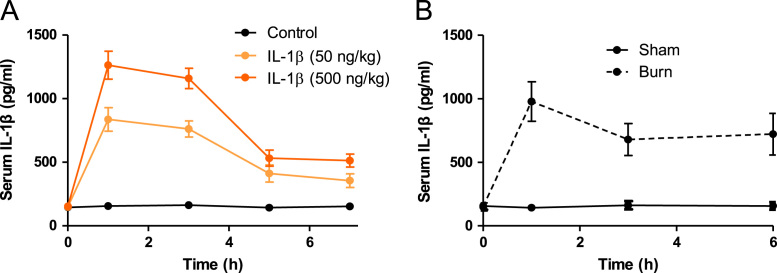
Serum IL-1β in control mice with a single injection of IL-1β (A) or burned mice (B). *n* = 5.

**Fig. 6:**
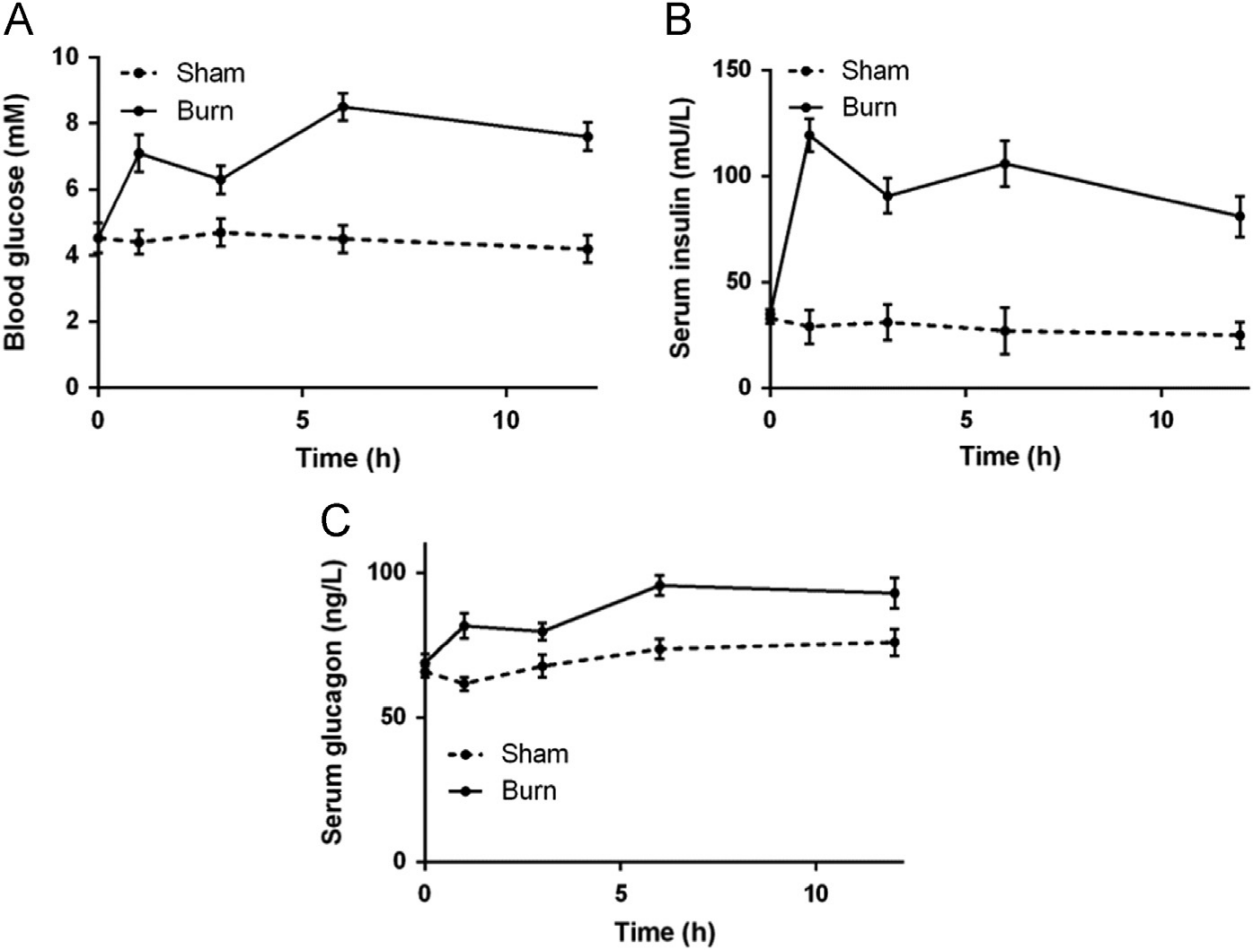
Postburn blood glucose (A), serum insulin (B) and serum glucagon (C) in burned mice. *n* = 5.

**Fig. 7:**
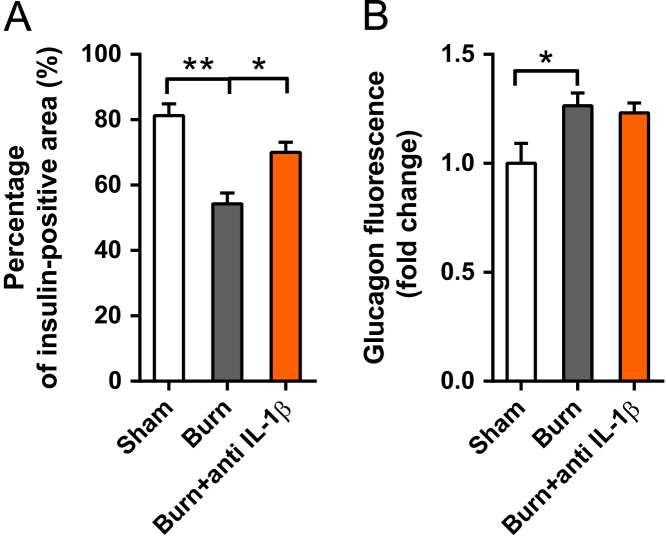
Quantitative results of insulin-positive area (A) and glucagon fluorescence (B) in pancreatic islets from mice with severe burns (*n* = 21 from 3 mice).

A total of 1643 blood samples were collected, of which 1358 blood samples were from burn patients and 285 blood samples were from control patients [1]. Linear regressions for blood glucose with serum insulin, glucagon and glucocorticoid as covariates in control and burn patients were conducted (Fig. 1 and Table 1, Table 2). Levels of serum proinflammatory cytokines were detected in both control and burn patients (Fig. 2). Correlations between serum insulin and serum proinflammatory cytokines were analyzed (Fig. 3). A tissue expression profile of IL-1 receptor was conducted in mice (Fig. 4). Serum IL-1β was detected in control mice with a single injection of IL-1β and burned mice without treatment (Fig. 5). Postburn blood glucose, serum insulin and serum glucagon in burned mice are shown in Fig. 6. Statistical results of insulin-positive area and glucagon fluorescence in pancreatic islets from mice with severe burns are shown in Fig. 7.

**Table 1:** **Multiple linear regression analysis for blood glucose in burned patients**.

**Independent variable**	**ASBI≤6**			**6 < ASBI < 10**			**ASBI ≥ 10**		
	**Regression coefficient**	**SE of regression coeffienct**	***P***	**Regression coefficient**	**SE of regression coeffienct**	***P***	**Regression coefficient**	**SE of regression coeffienct**	***P***
**Intercept**	5.909	0.155	.000	5.352	0.655	.000	7.244	1.014	.000
**Serum insulin (mU/L)**	0.003	0.009	.714	-.042	0.014	.004	−0.097	0.020	.000
**Serum glucagon (ng/L)**	0.006	0.002	.001	.010	0.002	.000	0.017	0.003	.000
**Serum glucocorticoid (μg/L)**	0.002	0.001	.011	.017	0.002	.000	0.010	0.003	.006

**Table 2:** **Multiple linear regression analysis for blood glucose in control patients**.

**Independent variable**	**Control patients**		
	**Regression coefficient**	**SE of regression coeffienct**	***P***
**Intercept**	2.445	0.537	.000
**Serum insulin (mU/L)**	0.097	0.018	.000
**Serum glucagon (ng/L)**	0.004	0.007	.553
**Serum glucocorticoid (μg/L)**	0.014	0.003	.000

## 2 Experimental design, materials and methods

### 2.1 Study population

Human study was approved by the institutional review board of Fourth Military Medical University and registered at www.chictr.org.cn (ChiCTR-OOB-15006420) as described previously [1]. Once admitted, the Abbreviated Burn Severity Index (ASBI) was calculated [2] and blood samples were randomly collected. Patients admitted to the same department receiving skin plastic operation were enrolled as the control. Blood variables were detected and multiple linear regressions were conducted as described previously [1].

### 2.2 Animal experiments

Animal experiments were performed according to the National Institutes of Health Guidelines for the Use of Laboratory Animals and were approved by the Fourth Military Medical University Committee on Animal Care. After being anesthetized, 30% total body surface area (TBSA) and full-thickness burn model was conducted as described previously [1], [3]. IL-1β antibody (200 mg/kg) (R&D systems, MN) was intraperitoneally administered immediately after burns. Immunofluorescence and other experiments were described previously [1].

### 2.3 Statistics

Continuous variables were compared by Mann–Whitney U or Student׳s t-test as appropriate. Linear relationships between variables were tested by Pearson׳s correlation coefficient. Multiple linear regression analysis was performed to evaluate independent contributions of the studied variables to blood glucose. The results are expressed as means ± SEM unless noted otherwise. A p value of less than 0.05 was considered statistically significant.
